# A systematic review of recruitment and retention of ethnic minorities and migrants in obesity prevention randomised controlled trials

**DOI:** 10.1038/s41366-024-01545-z

**Published:** 2024-06-04

**Authors:** Nidhi Wali, Md. Nazmul Huda, Timothy Gill, Julie Green, Andre M. N. Renzaho

**Affiliations:** 1 School of Social Sciences, Humanitarian and Development Research Initiative (HADRI), Western Sydney University, Sydney, NSW 2751 Australia; 2 Discipline of Psychiatry and Mental Health, School of Clinical Medicine, University of New South Wales, Sydney, NSW 2052 Australia; 3 Charles Perkins Centre, University of Sydney, Sydney, NSW 2050 Australia; 4 Murdoch Children’s Research Institute, Parkville, VIC 3052 Australia; 5 Department of Paediatric, University of Melbourne, Parkville, VIC 3052 Australia; 6 School of Medicine, Western Sydney University, Campbelltown, NSW 2560 Australia; 7 Translational Health Research Institute (THRI), Western Sydney University, Campbelltown, NSW 2560 Australia

**Keywords:** Risk factors, Epidemiology

## Abstract

**Background:**

Participants’ recruitment and retention into community-based interventions can be challenging, especially in research involving ethnic minorities and migrants. Despite known challenges, there are limited reviews that probe recruitment and retention strategies involving ethnic minorities and migrants in the Organisation for Economic Cooperation and Development (OECD) countries. This systematic review aimed to measure recruitment and retention rates and identify the barriers and facilitators to effective recruitment and retention of ethnic minorities and migrants in community-based obesity prevention Randomised Control Trials (RCTs) in OECD countries.

**Methods:**

This review was conducted according to the Preferred Reporting Items for Systematic Reviews and Meta-Analyses (PRISMA) guidelines. Five databases (CINAHL, Cochrane, Embase, Medline and PsychInfo) were searched from January 2000 to March 2022, in addition to Google and Google Scholar. Methodological quality and risk of bias were assessed, and pooled analysis and meta-ethnographic analysis were conducted on the included studies.

**Results:**

Twenty-five studies were included in the review. The pooled analysis found a 64% rate of recruitment of ethnic minorities in RCTs, with a retention rate of 71%. Key facilitators identified were—use of multiple communication channels, incentives, recruiting community champions, participant convenience and employing culturally sensitive strategies. Key barriers to participation were limited access to study sites, time constraints, limited trust, perceived fear, and anxiety.

**Conclusion:**

Findings suggest the importance of undertaking culturally appropriate recruitment and retention strategies to minimise barriers and facilitate effective recruitment and retention of low-income ethnic minorities and migrants in community-based research.

## Introduction

Recruitment and retention of ethnic minorities and migrant populations into community-based obesity prevention trials continues to be a challenge [1, 2]. This is due to multiple factors, including work-and family-related barriers, limited awareness of existing programmes [3], competing priorities, fear, mistrust [4, 5], participants’ time constraints, inadequate transport facilities, and increased mobility of participants [6]. Moreover, a culturally incongruent discussion about informed consent to participate and limited recruitment of investigators and staff from similar ethnic backgrounds can hinder the effective recruitment and retention of participants in community-based obesity prevention trials [5]. Approximately 50–70% of community-based obesity prevention trials are unable to meet the target estimated sample size because of low recruitment and retention of participants [7]. Such poor and slow recruitment and participant retention in community-based obesity prevention trials may generate inconclusive findings, affect the delivery of interventions, and increase a trial’s cost [1].

To overcome these barriers some strategies have shown successful recruitment and retention of participants from ethnic minorities into community-based obesity prevention trials. Evidence suggests that community outreach and in-person on-site recruitment at recreational centres, schools, and faith-based organisations such as churches, temples and mosques, are successful strategies for including participants in interventions [2]. Furthermore, a combination of strategies involving consistent follow-up and frequent communications by, for example distributing flyers and posters in multiple languages, radio messaging, texting, phone calls, emailing and sending letters and reminders, are required to enhance participants’ recruitment and retention in interventions [8, 9]. Gaining support from community leaders [10], community-based recruitment, recruitment of additional bilingual staff, contacting eligible potential participants with trained staff, flexible timings (weekends and evenings), and a suitable study site are also reported as important recruitment and retention strategies [8]. Moreover, building trust with participants [11], collaborating and developing relationships with communities [4, 12], and friend referral [9] may play a critical role in facilitating recruitment and retention of participants into studies. Additionally, funding community-based interventions [12], providing financial incentives to assist with the costs of participation, for example, travel [9, 13] and keeping study participants engaged through newsletters and social gatherings [3] may demonstrate commitment in increasing participants’ recruitment and retention into interventions. These strategies may guide researchers and staff towards effective recruitment and retention of study participants into community-based obesity prevention interventions.

To date, there are limited systematic reviews that brings together evidence to understand common barriers and facilitators of recruitment and retention of participants from ethnic minorities and migrant populations in community-based obesity prevention trials. A 2021 systematic review identified barriers (e.g., time constraints, limited understanding of clinical trials, consent complexity, inadequate transportation and limited childcare benefits) and facilitators (e.g. benefits for participants and others, financial incentives, friend referral, support, and recommendations from medical practitioners) to children’s participation in obesity interventions in the United States, Australia, Europe, and Canada [7]. Other reviews on the barriers and facilitators to ethnic and migrant populations’ participation in interventions were conducted in the United Kingdom [14] and the US [5, 15, 16]. However, these systematic reviews did not include ethnic minorities and migrants from most countries of the Organisation of Economic Cooperation and Development (OECD) and did not explore the barriers and facilitators of their effective recruitment and retention specifically in community-based obesity prevention trials. Therefore, there is limited understanding about the barriers and facilitators of recruitment and retention of ethnic minorities in community-based obesity prevention randomised control trials (RCTs). This systematic review aims to address the gaps in previous research through identifying, collating, appraising, and synthesising available literature on the recruitment and retention rates of ethnic minorities and migrants, and the related barriers and facilitators to effective recruitment and retention of ethnic minorities and migrants in community-based obesity prevention RCTs in OECD countries. Our findings will inform practice and support the development of empirical studies and programme evaluations, and the execution of community-based obesity prevention RCTs designed for ethnic minorities and migrants in OECD countries.

## Methods

A systematic review was conducted according to the Preferred Reporting Items for Systematic reviews and Meta-Analyses (PRISMA) guidelines [17]. The protocol was registered with The International Prospective Register of Systematic Reviews (PROSPERO: ID: CRD42022326690).

### Search strategy

A comprehensive search of peer-reviewed articles from five computerised bibliographic databases using relevant MeSH terms or subheadings of key words was conducted. The databases searched were CINAHL, Cochrane, Embase, Medline and PsychInfo. Google and Google Scholar were searched to identify any additional studies. Search terms were adapted to meet the requirements of different databases. Supplementary Table 1 provides the search terms used.

### Inclusion criteria

The Participants, Intervention, Comparison, and Outcomes (PICO) framework was used to guide our eligibility criteria, provided below [18]. This review included randomised controlled trials (RCTs), cluster randomised trials or quasi-experimental study designs for obesity interventions for minority communities, including adults, adolescents, and children in OECD countries [19]. Studies published in English from January 2000 to March 2022 were included in this review. The year 2000 was chosen as the base year due to the rapid rise in migration globally [20]. Studies were included if they were peer-reviewed and explored the barriers and/or facilitators of recruitment and/or retention of ethnic minorities, migrants and/or indigenous populations in community-based obesity prevention RCTs. This study defined recruitment as a process of finding and enrolling eligible participants in an RCT. Retention is defined as an RCT’s capacity to keep participants enrolled and engaged during its entire duration [21]. Ethnic minorities are groups of individuals who are distinct from majority populations regarding race, ethnicity, nationality, culture, language, and religion. Migrants are any individuals who changed his/her usual country of residence for various reasons, including work, education, family reunification, or seeking asylum [22].

Study eligibility criteria using the PICO framework.

**Table:** 

PICO	Included	Excluded
Participants	Migrants, refugees, ethnic minority groups, asylum seekers and indigenous populations of all age groups, including adults, adolescents and children (5–11 years) and those residing in OCED countries.	Literature that did not highlight the barriers and/or facilitators of recruitment and/or retention of ethnic minorities, migrants and/or indigenous populations in community-based obesity prevention programmes.
Interventions	• Randomised controlled trials; Cluster randomised trials or quasi-experimental studies obesity interventions. • Study design/objective must be an obesity prevention and/or obesity related intervention	
Comparison	General populations	
Outcomes	Obesity, Body mass index, overweight, adiposity, weight gain, bodyweight, and weight management	

### Exclusion criteria

Studies that did not highlight the barriers and/or facilitators of recruitment and/or retention of ethnic minorities, migrants and/or indigenous populations in community-based obesity prevention RCTs were excluded. Studies that met the inclusion criteria but provided scant data were excluded, determined via discussion between two researchers (MNH and NW). This review also excluded grey literature documents, review papers, conference presentations, protocols and studies not published in English.

### Screening

The retrieved articles were imported from each database into an Endnote library. Duplicates were removed in Endnote before screening in MS Excel. Title and abstract of the identified studies were screened by two authors in MS Excel (MNH and NW). Reasons for exclusion were recorded at the full-text stage (i.e. no information on outcomes, barriers and facilitators of effective recruitment and retention of ethnic minorities and migrants in community-based obesity prevention programmes in industrialised countries). Full texts of all studies were reviewed by the second author (MNH) and a sample of 30% were reviewed by the primary author (NW). Any discrepancies between two reviewers were resolved through discussion with a third reviewer (AR).

### Data extraction

MNH and NW conducted the data extraction of included studies in MS Excel. Quantitative and qualitative data from mixed methods studies were extracted separately (see Table 1). Data extracted from studies included general information (authors, publication year, country, research design, data analysis, participants’ ethnicity, sample size, and main findings on barriers and facilitators of effective recruitment and retention of ethnic minorities and migrants in community-based obesity prevention programmes). Two authors (MNH and NW) reviewed the entire data extraction file to ensure accuracy and consistency.

**Table 1:** Descriptions of studies included in the review.

Authors, publication year	Country	Sample size and characteristics	Main findings				QA score*
			Recruitment (%)	Retention (%)	Reasons for low recruitment and retention	Facilitators of effective recruitment and retention	
Marquez et al. (2020) [30]	USA	*N* = 438 Low-income Latino men and women Age: ≥50 years BMI 31.9 kg/m^2^	54.9%	66.67%	• Lack of trust • Language • Poor health: acute and chronic illnesses • Lack of safety • Poor transport • Work and care commitments • Low health and programme literacy • Inaccurate phone numbers • Others: Travel and holiday break, bad weather	• Established community relationships. • Recruitment through community centres and local organisations • Community health workers (CHWs) to assist with recruit, identify and screen potential participants, schedule appointments, and support data collections. • Physicians involved in distribution of flyers to disseminate study materials. • Reimbursing travel expenses.	Med, 6
Griffin et al. (2019) [46]	UK	*N* = 90 • Fathers (aged 18–65 years) from minority ethnic groups who were obese/overweight, and children aged 4–11 years	61%	63%	• Poor communication • Unable to find a suitable time and venue for all stakeholders • Work commitments • Limited time for content delivery • Weather: winter	• Local recruitment of health trainers. • Adapt delivery of courses based on available time and venue suitable to participants.	High, 9
DeFrank et al. (2019) [8]	USA	*N* = 89 • African American and Latino children (ages 9–12 years) and their parent/guardian, low-income neighbourhoods	62%	84.3%	• Socio-environmental factors • Poor health and programme literacy	• Incentives given to participants: round-trip metro cards, $15 gift card at the first visit; $25 gift card at the second visit. • Retention strategies: assigned trained study staff to dyads for the study period. • Family-friendly experience and interactions with study staff with no explicit rule around bringing additional family members to study visit. • Flexibility to continue participation: virtual participation options for completing second and third study visits. • Provided a hospitality room and greeted by trained study staff with toys, food and beverages and had a seating area.	High, 8
Cui et al. (2019) [2]	USA	*N* = 1745 • Low-income racially diverse parent–child dyads	Not reported	Not reported	• Low participant interest • Poor health and programme literacy • Work and caring commitments, time conflicts • Poor transport • Cultural barriers • Inaccurate phone numbers	• Established community relationships. • Culturally sensitive staff • Flexible schedule and locations • Financial incentives to participate.	High, 7
Srivastava et al. (2018) [31]	USA	*N* = 13 • African American children and adults	23.4%	40%	• Work and caring commitments, time conflicts • Poor transportation • Loss of employment and housing	• Multiple reminder phone calls and letters prior to each group visit. • Regular contact with participants.	Low, 3
Metayer et al. (2018) [9]	USA	*N* = 406 • New immigrant mothers (in the US for 10 years or less) and children from Brazil, • Latin America, and Haiti	38%	Not reported	• Cultural barriers • Immigration status	• Building trust and community organisation partnerships. • Posters and flyers. • Media announcements. • Church outreach. • Participant referrals.	High, 7
Heerman et al. (2018) [32]	USA	*N* = 117 • Low-income, Hispanic and Latino minority preschool children	Not reported	Not reported	Not reported	• Multiple methods of contacts. • Culturally sensitive staff. • Regular contact with participants. • Financial incentives to participate. • Flexible schedule and locations.	High, 7
Dressel et al. (2018) [33]	USA	*N* = 49, • Low-income Latino and African Americans, overweight, or obese adults	77.5%	Not reported	• Low recruitment when women recruited men vs. women recruiting women; vice versa • Not having childcare support	• Recruitment flyers at local community-based clinics and organisations. • Conducted outreach at community events, and personally based on past interests. • Materials translated to participants language. • Bilingual staff.	Med, 5
Crespo et al. (2018) [34]	USA	*N* = 390, • Overweight and obese Latino children	78.16%	67.7%	• Delays in enrolment.	• Personal phone calls for recruitment.	High, 7
Lynch et al. (2017) [35]	USA	*N* = 269, • Low-income African • Americans from community clinics of a large, urban public hospital system.	44.6%	78.44%	• Lack of trust	• Recruitment, both in-person and telephonic, conducted from the patients’ own clinic (i.e., the telephone number on caller ID was familiar to patients as it came from the clinic). • Flexible schedule and locations. • Staff from similar cultural background. • Participants provided extensive information about the study prior to participation. • Transport cost reimbursed. • Financial incentives to participants.	High, 9
Bernstein et al. (2017) [36]	USA	*N* = 49, • Latino and African Americans aged 18–69 years with < days physical activity per week and BMI ≥ 25.0.	94.24%	53.06%	Not reported	• Rescheduling of biking rides. • Distributing flyers via community health ambassadors.	High, 7
Pekmezi et al. (2016) [37]	USA	*N* = 84, • Low-income African American women aged 50–69 years	45.41%	Not reported		• Financial incentives to participants. • Advertising on local radio stations and attendance at local churches and face-to-face recruitment by an experienced recruitment.	High, 8
Daly et al. (2016) [38]	USA	*N* = 47, • Latino females with14–17 years of age with a BMI >90th percentile	78.72%	61.0%	• Perceived fear of peer reactions/stigma • Not being ready to engage in the programme	Not reported	Med, 5
Coday et al. (2016) [39]	USA	*N* = 330 • Non-Hispanic/Latino Americans (18–35 years) with BMI < 40 kg/m^2^ who were overweight and smoked 18.4 cigarettes per day.	10.67%	Not reported	Not reported	• Mass mailings of postcards to age-appropriate persons as identified by driver’s license. • Programme advertisement on mass media (television, radio, newspaper), social media (trial Facebook page), internet (Google ads), and use email addresses.	Med, 6
Rosas et al. (2015) [40]	USA	*N* = 207, • Latinos with a BMI of 30–60 and one or more heart disease risk factors	53.0%	Not reported	Not reported	• 100 h of in-depth training of bilingual and bicultural staff before implementation. • Weekly staff debriefing sessions. • External evaluation of quality of intervention.	High, 8
Koniak-Griffin et al. (2015) [41]	USA	*N* = 223, • Latino women aged 35–64 years, predominantly with low income and ≤8th grade education.	77.43%	87.0%	Not reported	• The frequent contacts of promotor as with the women facilitated high retention, as they knew when and where participants moved.	High, 8
Cruz et al. (2014) [10]	USA	*N* = 1879, • Rural American Indian and Hispanic children	Not reported	Not reported	• Lack of trust • Long travel distances • High staff turnover	• Involving recruiting community champions to assist with the project, providing incentives, telephone reminders, increased site visits and over-scheduling of interviews, assigning one dedicated person to maintain contact. • Use of bilingual interviewers. • Face to face communicating in-with families. • Use of suitable language. • Flexible location and times.	High, 7
Anderson et al. (2014) [42]	USA	*N* = 38, • Low-income African American and Mexican American mothers of kindergartner	82.61%	Not reported	Not reported	• Worksite support to participate in the intervention for school-based interventions.	High, 8
Nicholson et al. (2011) [13]	USA	*N* = 191, • Low-income African American families, non-Hispanic and Hispanic.	Not reported	64%	No barriers to enrolment	• Piloting procedures. • Frequent team reporting. • Flexible with location and times, budget. • Financial incentives to participants. • Frequent contact with participants. • Involvement of clinical staff. • Coordinated effort between the research team and the infrastructure support at the clinical sites, and project branding. • Dedicated phone line.	High, 7
Vincent et al. (2013) [43]	USA	*N* = 58, • Spanish-speaking adults of Mexican origin	63.74%	92.6%	• Immigration law and status: fear and anxiety • Language barriers • Low health literacy • Limited access to primary care providers • Economic constraints • Limited transportation • Fear of participation	• Bilingual advertised materials visible areas of participating community health clinics, recruiting bilingual research team, using university communication channels (e-mail list serves). • Weekly reminder phone calls to participants • Weekly homework assignments to participants enhanced engagement	High, 7
Boudreau et al. (2013) [44]	USA	*N* = 41, • Latino children, aged—12 years with BMI _85%, in low-income community	79.0%	67.0%	• Socio-environmental factors Poor health and programme literacy	• Incentivised to participate: $15 gift card at the first visit; $25 gift card at the second visit.	High, 8
Warner et al. (2013) [3]	USA	*N* = 365, • Hispanic, non-Hispanic Black	77.0%	86.0%	• Difficulty in maintaining a large pool of possible participants for contact • Long waiting in receipt of provider approval • Inability to reach participants, frequent missed appointments, and missed follow-up visits for participants inactive in the intervention arm	• Establish robust relationships with clinic staff. • Transport vouchers. • Flexible with the time: night and weekend appointments. • Enhanced participant engagement via newsletters and social gatherings. • Patient self-referral, and friend’s referral. • Advertisements in participants’ language in newspapers, and prepaid phones for researchers.	High, 7
Kumanyika et al. (2005) [45]	USA	*N* = 237, • Low-income African Americans aged 25–70 years with a BMI 30–50 kg/m^2^	54.0%	36.71%	Not reported	• Appropriate space for data collection.	High, 8
Marshall et al. (2021) [47]	Australia	*N* = 163	95%	78%		• Programme delivery staff to be culturally sensitives and adaptive. • In-language resources and individualised bicultural nurse support by telephone for supporting culturally and linguistically diverse migrant families with infant feeding. and active play.	
Lindsay et al. 2021 [11]	USA	*N* = 233, Brazilian immigrants	NA	NA		• Direct recruitment methods in combination with snowball sampling and social media were effective. • Staff’s understanding of the sociocultural context along with linguistically and culturally sensitive recruitment strategies tailored to meet the needs of the community.	

QA score: Quality Assessment score: ‘High quality’ [7–9]; medium [4–6]; low (0–3).

### Methodological quality and risk of bias

Methodological quality and risk of bias were independently assessed by two authors (MNH and NW) using the CASP tool for RCTs [23] and the Cochrane risk of bias tool [24]. Nine criteria of the CASP tool were used to assess the methodological quality [23]. Studies with scores from 7 to 9 were considered ‘high’ quality, scores from 4–6 were ‘medium’ quality and 0–3 were classified as ‘low’ quality (Supplementary Table 2). The Cochrane risk of bias tool determines how well a study has addressed potential biases in its design and analysis [24]. The risk of bias of included studies was assessed using six domains of the Cochrane risk of bias tool (Supplementary Table 3). Discrepancies were discussed by both reviewers (MNH and NW), with a third reviewer (AR) brought in to reach a consensus for any disagreements.

### Data synthesis

Quantitative studies included in the review varied in study design, methods, definitions and measurement of outcomes and explanatory variables. Although a pooled analysis of proportions was performed to combine the results of included studies on participants’ recruitment and retention in obesity prevention RCTs, a meta-regression was not possible due to inconsistent reporting or marked clinical heterogeneity in study populations, interventions, and the outcomes studied [25]. The “metaprop” Stata 9 (version 16.0, StataCorp, College Station, TX, USA) command was used to generate forest plots for the ethnic minority and migrant share of the sample size and the proportion of retention (those in the intervention who completed it). Each forest plot shows the proportion and associated 95% confidence intervals (CI), and corresponding weight. A test of heterogeneity showed a high level of inconsistency (*I*^2^ > 50%), hence a random-effect model was used for the pooled analysis [26]. Sensitivity analyses were conducted to examine the effect of outliers comparing the pooled prevalence before and after removal of one study at a time [27].

Additionally, a meta-ethnographic approach was used to synthesis evidence of included studies [28], this approach is increasingly being recognised to synthesise data in reviews [29]. The meta-ethnographic approach involved four stages: (1) identifying metaphors and common findings by re-reading the studies to gain familiarity within the data and identify themes and patterns in each study; (2) determining how the study findings were related by comparing the thematic analysis of all studies: (3) reciprocal translation of studies by comparing the themes of all studies, this included comparison and matching of themes across papers to ensure that the key themes across studies are captured; and lastly (4) synthesising translations which form a line of argument for the description of findings. This approach allowed to identify relationships between studies, which led to a better understanding of effective recruitment and retention strategies of ethnic minorities and migrants in community-based obesity prevention trials. It allowed to identify common factors across studies and reciprocal translation of studies to inform findings around recruitment and retention strategies of ethnic minorities and migrants.

## Results

**Fig. 1:**
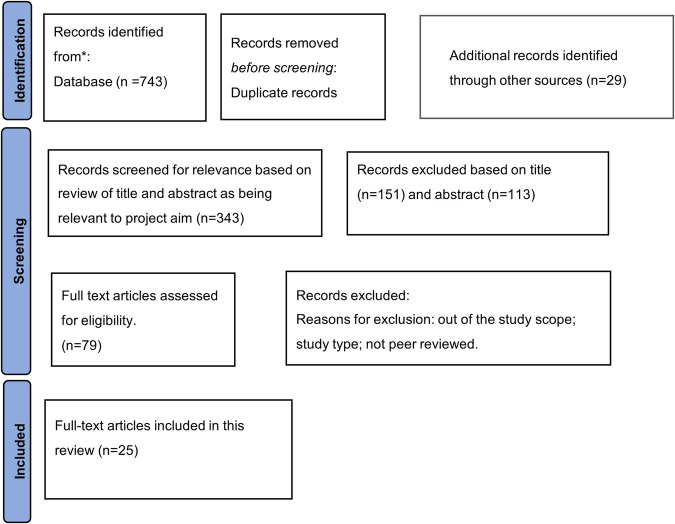
Systematic search results in PRISMA flow chart.

In total 772 records were identified from five databases, Google, and Google Scholar searches. After removing duplicates and conducting the screening of titles, abstracts, and full texts, 25 studies were identified for inclusion in this systematic review. The reasons for excluding studies were: (1) not conducted in OECD countries, (2) not peer-reviewed, (3) not an RCT, and (4) full-text not available. Search results at each stage of the review process are illustrated in Fig. 1, using the PRISMA diagram.

### Characteristics of included studies

This review included 25 studies on recruitment and retention of ethnic minorities in obesity prevention RCTs. Of the 25 studies, majority of the studies were conducted in the USA (*N* = 23) [2, 3, 8–10, 13, 30–45] except 1 that was conducted in UK [46] and 1 in Australia [47]. Seven RCTs were exclusively conducted among Latino Americans [30, 34, 38–41, 44], 4 among African Americans [31, 35, 37, 45], and 1 among Mexican descent immigrants in the USA [43] and 1 among Brazilian immigrants in the USA [11]; 3 RCTs combined Latino and African American population [8, 33, 36], 1 combined Hispanic and Latino population [32] and 1 combined Hispanic and non-Hispanic black population [3]. The remaining studies were a combination of populations from different ethnic backgrounds [2, 9, 10, 13, 42, 46]. The studies used various statistical techniques for data analysis, including descriptive statistics (proportion, percentage, mean, interquartile range, standard deviation), inferential statistics (Chi-square, Fisher’s exact tests, *t*-test, ANOVA, Wilcoxon and Kruskal–Wallis tests, regression analysis). Few studies did not mention any analytical techniques.

### Recruitment and retention of ethnic minorities in obesity prevention RCTs

**Fig. 2:**
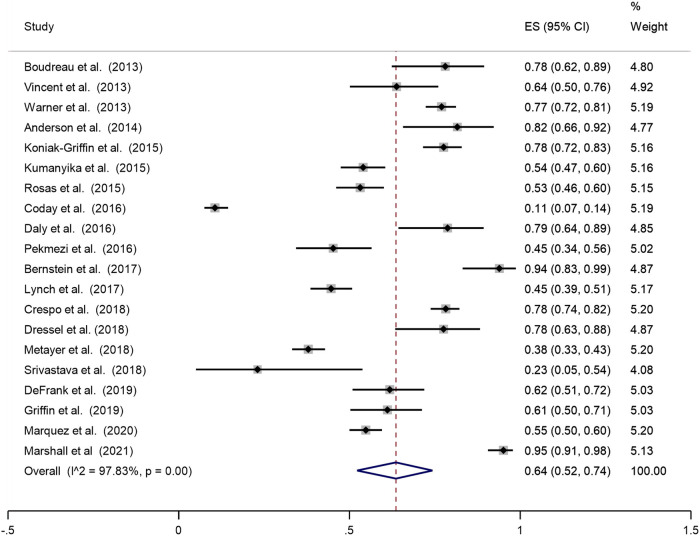
Pooled analysis of the proportion of the sample that is ethnic minorities and migrants.

**Fig. 3:**
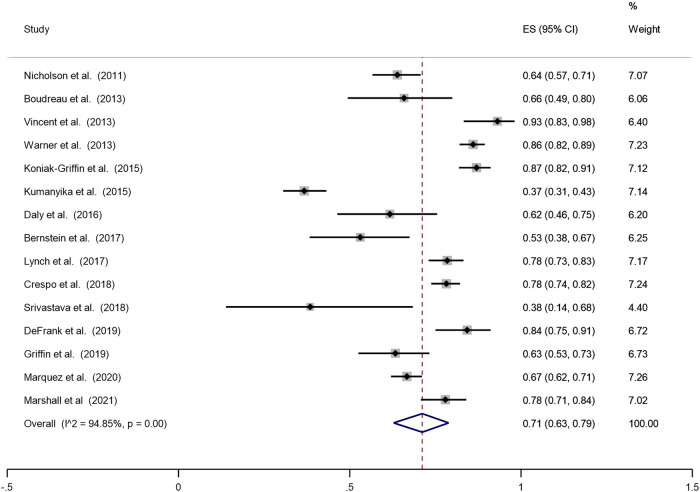
Proportion of ethnic minority and migrants who completed the programme.

This review found that the pooled proportion of recruitment of ethnic minorities in obesity prevention programmes and trials was 64% (Fig. 2). Of those recruited, the pooled proportion of retention of ethnic minorities in obesity prevention programmes and trials was 71% (Fig. 3).

### Barriers to effective recruitment and retention

The meta-ethnographic analysis of studies included in this review provide a range of barriers to the effective recruitment and retention of ethnic minorities and migrants in community-based interventions in OECD countries. These findings are summarised below.

### Barriers related to study characteristics

Studies reported study characteristics contributing to the recruitment and retention of ethnic minorities in RCTs, including intervention types, dose (duration, frequency), and involvement types. For example, studies discussed the different types of interventions aimed at recruiting and retaining participants, such as lifestyle and behavioural interventions [30, 34, 41, 46], interactive group classes on lifestyles [44], nutrition education and counselling by a dietitian [31], curriculum on nutrition and physical activity [9], bicycling [33, 36], exercise [37], behavioural weight management intervention [39], and weight-loss interventions [40]. Types of involvement (physical, face-to-face delivery of interventions [37, 44] telephone and online delivery of intervention [44, 46], also affected participants’ recruitment and retention in RCTs. The duration of the RCTs varied; one for 3 years [2], one for 2 years and 7 months [41], three ran for 2 years [3, 9, 40], one for 18 months [45], and three for 1 year [31, 33, 36]. The rest lasted for less than 6 months [36, 43, 48]. Limited duration of intervention of less than 6 months contributed to decreased participants’ recruitment and retention [46].

### Limited access to study sites

Three studies with ethnic minorities, such as, American Indians [10], Latinos [30], and African Americans [31], highlighted the limited access to study site as a reason to not to be part of the recruitment process. Their participation was impacted by long travel distances [10] and transportation problems [2], including limited transport, problems with cars and high travel costs [11, 31, 41].

### Time constraints

Time conflict was commonly reported across several ethnic minorities and migrant communities (e.g. Latinos, Brazilian immigrants, African Americans, etc.) [2, 11, 31, 46, 47]. These time conflicts were due to participants’ commitments to family activities, jobs [2, 46], childcare, housing and limited personal time [47], affecting their participation [31]. Participants’ limited availability of time in the afternoon and evening due to children’s after-school activities, work commitments and children’s meals and bedtimes also impacted their participation [46].

### Lack of trust

Four studies mentioned a lack of trust as a barrier to effective recruitment and retention of ethnic and migrant communities [2, 10, 30, 35]. According to these studies, participants had a history of distrust with researchers particularly medical care and research, including previous experience of disrespect, poor quality of medical care and researchers not following through commitments. These studies established trust with communities through repeated contacts, listening, shared learning, bi-directional communication, following through on commitments and mutual respect.

### Perceived fear and anxiety

Perceived fear and anxiety were a barrier reported in three US studies conducted among Latinos [38], Spanish-speaking adults of Mexican origin [43], and Brazilian immigrants [11]. Many ethnic migrants, such as Mexican Americans [43] and Latinos [38], were afraid of participating in interventions due to fear and anxiety produced by strict anti-illegal immigration laws [43]. Fear of revealing information to researchers also discouraged the participants from taking part in the programmes [11].

### Other barriers to recruitment and retention

Other barriers to recruitment and retention included a lack of safety for participants with special needs such as walking difficulty [30], language barriers, low levels of health and intervention literacy [43], and perceived stigma [38]. The current review also found other barriers of participation-associated costs [11], difficulty in maintaining a large pool of potential participants for contact, and prolonged waits in receipt of provider approval [3].

#### Facilitators of effective recruitment and retention

**Fig. 4:**
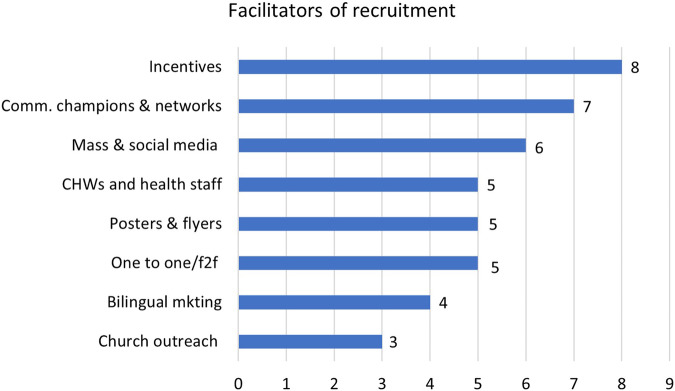
Facilitators of effective recruitment.

**Fig. 5:**
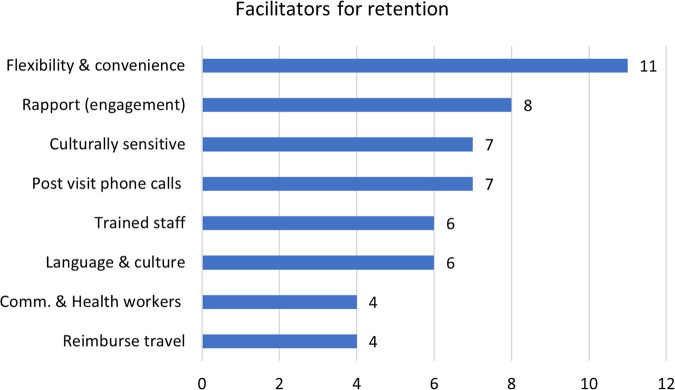
Facilitators of effective retention.

Facilitators of effective recruitment and retention of ethnic minorities and migrants in RCTs are summarised below. Figures 4 and 5 outline the most effective recruitment and retention strategies, study wise description of these is provided in Supplementary Table 4.

### Using multiple communication channels

The use of multiple communication channels, including emails, flyers, frequent contacts with participants and sending weekly reminders via telephone and mobile text, etc., were reported as essential to facilitate participants’ recruitment and retention. Twelve studies highlighted communication as a facilitator, these study participants included low-income Latino [30, 34, 41] and African Americans [13, 33, 36], Hispanic immigrants [3, 32], non-Hispanic/Latino Americans [39], mothers and children from Brazil, Latin America, and Haiti [9], Spanish-speaking adults of Mexican origin [43], rural American Indian and Hispanic children [10], and Brazilian immigrants in the US [11]. Moreover, university communication channels (e-mail lists) helped disseminate study information among potential study participants and facilitated their recruitment in research [43].

### Employing culturally sensitive strategies

Eight of the 25 studies conducted with low-income ethnic minorities and migrants living in the US [2, 10, 11, 33, 35, 40, 43] and Australia [47] documented culturally sensitive strategies (e.g. cultural competency, recruiting bilingual and culturally sensitive staff, translating recruitment and study materials into participants’ languages) as a critical facilitator of recruitment and retention of participants. For example, a study with Brazilian immigrants in the US found that staff member’s understanding of participants’ sociocultural context and the application of linguistically and culturally sensitive recruitment strategies helped meet the needs of participants and facilitated their successful recruitment and enrolment [11]. Bilingual staff who were culturally competent and sensitive to participants were able to effectively deliver study materials and presentations in the participant’s languages [2, 10, 33, 35, 40, 43]. Such culturally sensitive strategies increased participants’ recruitment and retention, in particular when bilingual and bicultural staff were provided with in-depth training before recruiting participants in health research [40].

### Incentives and compensation for participants

Monetary incentives (gift cards, recipe books, cash, reimbursements for transportation, parking, childcare) and compensation for participants after data collection helped reduce barriers to participants’ recruitment and retention with low-income and multi-ethnic communities [2], including African-American women [13, 37], children from Hispanic and Latino communities [32, 44] and rural American Indian and Hispanic children [10]. A US bases RCT reported that having an adequate budget helped provide a financial base for financial incentives to the participants [2]. For example, providing participants with $25 for the completion of the baseline assessment, $50 for the completion of assessments at weeks 12 and 24, and $25 for the completion of mini-assessments kept participants engaged in childhood obesity prevention and prevention studies [2].

### Participant convenience

As described in Table 1, participants’ convenience was one of the most common facilitators, appearing in 7 of the 25 included studies. The use of a dedicated phone [13], the time [10] and locations convenient to participants [2] and providing scheduling flexibility outside participants’ regular work for completing studies, addressed barriers to participants’ recruitment and retention in studies [7, 32, 36].

### Recruiting community leaders/local champions

Three RCTs, all conducted in the USA, reported recruiting local champions to contact potential participants, schedule interviews with them and recruit them for studies [2, 10, 30]. The largest study, which recruited rural American Indian and Hispanic children, found that recruiting community leaders, who hold a strong position in the community, can serve as a primary contact to assist with the project, including advocating the project and promoting its benefits [10]. The second RCT included participants from low-income, racially diverse parent–child dyads, reported that strengthening connections with the local community leaders and soliciting information from them helped retain the participants [2]. Building trust with the community leaders and strengthening relationships with them helped disseminate study materials and sustained their participation in the interventions [30].

### On-site/in-person recruitment

Studies with low-income African Americans [35], Brazilian immigrants born in the US [11], and rural American Indian and Hispanic children [10] that had on-site/in-person recruitment enhanced participant engagement. For instance, Lindsay et al. [11] found that on-site in-person recruitment at church (e.g., mass and other church events), and private social and community events (e.g., health and cultural fairs), were effective recruitment strategies in health research.

## Discussion

### Summary of key findings

This systematic review reported on the recruitment and retention of ethnic minorities in community-based obesity prevention RCTS with the pooled proportions of recruitment and retention at 64% and 71% respectively. Evidence on recruitment and retention rates is limited and varied across literature. For instance a systematic review reports considerable variability in recruitment rates (median 66.4%; interquartile range = 42.7–85.2) and retention rates (median 80.5%; IQR = 68.5–89.5) [49]. While our findings indicate a high pooled proportion of recruitment of 64%, this could be due to successful strategies that are culturally sensitive and based on participants convenience. Our findings suggest of those recruited, the pooled proportion of retention of ethnic minorities in obesity prevention programmes and trials was 71%, which further indicates the success of strategies employed for retention of participants.

Our findings revealed multiple barriers to effective recruitment of ethnic minority and migrants into obesity prevention programmes, including African Americans, American Indians, Mexican Americans, Hispanic Latinos, and Brazilian immigrants in the US, Arabic- and Chinese Australian and young adults living in UK, Australia, and Ireland. These included limited access to study sites, time constraints, limited trust, perceived fear, and anxiety. The identified barriers in this systematic review are consistent with those identified previously. For instance, Clayton et al. found lack of transportation made accessing the study sites difficult to access and time constraints to get to the clinic amidst other commitments [7]. Similarly, George et al. revealed mistrust and consequent fear of participation as a key barrier of recruitment and participation of migrants and ethnic minorities in the US in clinical research [5].

Our review findings highlight facilitators of effective recruitment and retention of ethnic minorities in community-based obesity prevention programmes. These included use of multiple communication channels, incentives, recruiting community champions, participant convenience and employing culturally sensitive strategies. Research suggests community outreach and in-person on-site recruitment at recreational centres, schools, and faith-based organisations such as churches, temples and mosques, as proven strategies for successful recruitment and ongoing participation in clinical research [2]. Similarly, convenience to participants such as flexible timings (weekends and evening), and a suitable study site also contributed to successful recruitment and retention of African American and Latino youth participants in research [8]. Our findings concur with research that highlights a combination of communication tools such as involving consistent follow-up and frequent communications by tools such as distributing flyers and posters in multiple languages, radio messaging, texting, phoning, emailing and sending letters and reminders, are required to enhance participants’ recruitment and retention [8, 9]. Financial incentives for participants is also an effective tool to improve retention of participants in research [5, 49].

### Application of multiple strategies together can facilitate effective recruitment and retention

Review findings suggest that using multiple strategies can help reduce barriers to participants’ recruitment and retention in studies and facilitate effective recruitment and retention of ethnic minorities and migrants in obesity prevention programmes, consistent with previous research [50]. Further our findings suggest that multiple strategies need to embed culturally competent and safe recruitment practices to attract and recruit ethnic minorities. Culturally competent recruitment practices promote a research environment that allows researchers to recruit effectively in cross-cultural situations [51], while culturally safe recruitment practices prioritise shared respects, knowledge reciprocity, and experience in a research environment that makes research participants feel socially, emotionally, and spiritually safe [52]. Israel et al. indicate that such practices should meet 8 criteria: (1) recognise the collective nature of ethnic minorities’ communities as a unit of identity (e.g. membership in a family, friendship network, or geographic neighbourhood); (2) build on strengths, resources, and relationships that exist within communities of identity (e.g. recognising, supporting, and expanding skills, assets, and social structures and social processes that enable community members to work together); (3) facilitate collaborative partnerships in all phases of the research implementation; (4) Integrate knowledge and action for mutual benefit of all partners; (5) promote a co-learning and empowering process that attends to social inequalities; (6) involve a cyclical and iterative process; (7) Address health from both positive and ecological perspectives; and (8) Disseminate findings and knowledge gained to all partners [53]. Implicit in these criteria is that ethnic minorities need to be well informed research participants and have the agency to make decision. This is why our findings emphasise the importance of multiple field‐based strategy and snowballing strategies that can work at different levels and help reduce participants’ barriers to participation in health research such as community mistrust of the research process, the need to culturally appropriate compensation, and overcoming language barrier and low level of literacy. Recruitment strategies might include use of flyers, newspaper advertisements, community outreach, snowball, and social media [54]. Retention strategies might include rapport with ongoing engagement, post-visit phone calls, fair compensation, flexibility, frequent reminders, and being respectful and sensitive [54]. Therefore, researchers can effectively recruit and retain participants by reducing barriers if they build rapport with participants via multiple communication channels, addressing their needs (e.g. flexibility in schedules and techniques, incentives) and recruiting them in person [55]. Employing bi-cultural staff and investing in the their training may further enhance their skills in recruitment [40] and increase recruitment and retention rates [55], thus increasing generalisability [56].

### Implications of recruitment and retention

Given that the success of a project largely depends on the effective recruitment and retention of participants, addressing the barriers whilst promoting facilitators can enhance ethnic minorities and migrants’ participation and retention in community-based obesity prevention programmes. As multiple social factors, including stigma [38], distrust [35], and limited health literacy [43], can hinder participants’ enrolment in research, providing staff with appropriate training on cultural sensitivity may enhance the participation of diverse ethnic and migrant communities. It is also equally important to provide ethnic minorities with relevant education and brief them about concerns. For example, an education session for African Americans may include a historical context of distrust and its related causes. During these sessions, participants can openly discuss the issues [5]. Moreover, allowing participants to ask questions about any concerns about the research process and protective measures for participants may address their fear and anxiety associated with their participation in the studies. Furthermore, when a clinical trial involves populations from low-income ethnic minorities and migrants, there may be a requirement to approach participants using various communication channels [43] and community champions [10], with scheduling flexibility [32] and incentives [37]. These efforts may help recruit and retention participants from low-income multi-cultural communities living in high-income countries in a culturally appropriate manner [5]. Such approaches may reduce the barriers to recruitment and retention and facilitate their effective participation in health research.

### Strengths and limitations

Our study has several strengths. First, to the best of our knowledge, this is the first study to systematically review the barriers and facilitators of effective recruitment and retention of ethnic minorities and migrants in community-based obesity prevention trials in OECD countries. Second, the application of a systematic approach [17] to search data from relevant scientific databases enabled the revealing most available papers on the subject. Third, we used a comprehensive list of search terms covering many ethnic minorities and migrants in OECD countries and conducted these searches across five electronic databases, Google and Google Scholar. Finally, we used rigorous and objective measures to assess for eligibility and evaluate for risk of bias. However, our review does have a few limitations. The review only included studies written in English due to time and resource constraints. Thus, this review may have missed some relevant papers published in other languages. Another limitation is that the review was limited to articles focusing on the barriers and facilitators of effective recruitment and retention of ethnic minorities and migrants in community-based obesity prevention trials in OECD countries. As a result, valuable accounts on the barriers and facilitators of effective recruitment and retention of ethnic minorities and migrants in community-based obesity prevention trials beyond OECD countries were excluded. Lastly, majority of the studies were conducted in the US, potentially limiting their representativeness for recruitment and retention practices across all OECD countries.

## Conclusion

Our systematic review of the peer-reviewed literature found a low recruitment rate and identified many barriers and facilitators of effective recruitment and retention of ethnic minorities and migrants in community-based obesity prevention programmes. However, most studies we reviewed were conducted with ethnic minorities and migrants living in the US. Our review points to the urgent need to conduct more research focusing on multicultural ethnic minorities living in other OECD countries. Our review suggests the importance of undertaking culturally appropriate multiple recruitments and retention strategies to minimise barriers and facilitate the effective recruitment and retention of ethnic minorities and migrants in community-based research.

### Supplementary information

Search terms

Quality Assessment

Risk of bias assessment

Factors for effective recruitment and retention

## Data Availability

All data generated or analysed during this study are included in this published article.
