# Supplementary material for: A systematic review of recruitment and retention of ethnic minorities and migrants in obesity prevention randomised controlled trials
Source: Int J Obes (Lond). 2024 Jun 4;48(8):1065–79. doi: 10.1038/s41366-024-01545-z (PMC11281904; doi:10.1038/s41366-024-01545-z)
Supplement: Supplementary file 1 — Search terms [file 41366_2024_1545_MOESM1_ESM.docx]

**Supplementary table 1.** Search terms used in this systematic review.

| Patient, Population or Problem | Migrant* (MeSH) OR refugee* (MeSH) OR population OR “population groups” (MeSH) “ethnic groups” OR “asylum seeker” OR minority OR “indigenous population”  AND  Barrier* OR facilitator* or enable* OR disenable* (no MesH)  AND  “community participation” (MeSH) OR participation OR “Voluntary admission” OR recruitment OR admission OR participation OR retention  AND |
| --- | --- |
| Interventions and comparisons | Intervention* OR “prevention intervention” OR program OR “Randomi?ed controlled trials” (MeSH) OR “Clincal trial” OR “Cluster randomi?ed trials” OR “quasi experimental” OR RCT OR “Difference in difference” OR “Nonequivalent control group design” OR “no-treatment control group design” OR “nonequivalent dependent variables design” OR “removed treatment group design” OR “repeated treatment design” OR “reversed treatment nonequivalent control group design” OR “cohort design” OR “post-test only design” OR “regression continuity design” OR “Regression discontinuity design” OR “Case-control design” OR “time-series design” OR “multiple time series design” OR “interrupted time series design” OR “propensity score matching” OR “instrumental variables” OR “Panel analysis”  AND |
| Outcomes | Obes* (MeSH) OR “Body Mass Index” (MeSH) OR BMI OR overweight OR adiposity OR “weight gain” OR bodyweight OR weight management |
