# Supplementary material for: A systematic review of recruitment and retention of ethnic minorities and migrants in obesity prevention randomised controlled trials
Source: Int J Obes (Lond). 2024 Jun 4;48(8):1065–79. doi: 10.1038/s41366-024-01545-z (PMC11281904; doi:10.1038/s41366-024-01545-z)
Supplement: Supplementary file 2 — Quality Assessment [file 41366_2024_1545_MOESM2_ESM.docx]

**Supplementary table 2.** Quality assessment of included studies

| **Author and year** | **Did the study address a clearly focused issue?** | **Was the assignment of participants to interventions randomised** | **Were all participants who entered the study accounted for at its conclusion?** | **Were patients, health workers and study personnel ‘blind’ to treatment?** | **Were the study groups similar at the start of the trial?** | **Aside from the experimental intervention, were the groups treated equally?** | **How large was the treatment effect?** | **Can the results be applied to the local population, or in your context?** | **Were all clinically important outcomes considered?** | **Total score** |
| --- | --- | --- | --- | --- | --- | --- | --- | --- | --- | --- |
| (Marquez et al., 2020) | 1 | 1 | 1 | 0 | 1 | 0 | 0 | 1 | 1 | 6 |
| (Griffin et al., 2019) | 1 | 1 | 1 | 1 | 1 | 1 | 1 | 1 | 1 | 9 |
| (DeFrank et al., 2019) | 1 | 1 | 1 | 0 | 1 | 1 | 1 | 1 | 1 | 8 |
| (Cui et al., 2019) | 1 | 1 | 1 | 0 | 1 | 1 | 0 | 1 | 1 | 7 |
| (Srivastava et al., 2018) | 1 | 0 | 0 | NA | NA | NA | 1 | 1 | 1 | 4 |
| (Metayer et al., 2018) | 1 | 1 | 1 | 0 | 1 | 1 | 0 | 1 | 1 | 7 |
| (Heerman et al., 2018) | 1 | 1 | 0 | 1 | 1 | 1 | 1 | 1 | 0 | 7 |
| (Dressel et al., 2018) | 1 | 1 | 0 | 0 | NA | 1 | 1 | 0 | 1 | 5 |
| (Crespo et al., 2018) | 1 | 1 | 1 | 0 | 0 | 1 | 1 | 1 | 1 | 7 |
| (Lynch et al., 2017) | 1 | 1 | 1 | 1 | 1 | 1 | 1 | 1 | 1 | 9 |
| (Bernstein et al., 2017) | 1 | 1 | 0 | 0 | 1 | 1 | 1 | 1 | 1 | 7 |
| (Pekmezi et al., 2016) | 1 | 1 | 1 | 1 | 1 | 0 | 1 | 1 | 1 | 8 |
| (Garcia et al., 2018) | 1 | 1 | 1 | 1 | 1 | 1 | 1 | 1 | 0 | 8 |
| (Daly et al., 2016) | 1 | 1 | 0 | 0 | 1 | 1 | 0 | 1 | 0 | 5 |
| (Coday et al., 2016) | 1 | 1 | 1 | 0 | 1 | 1 | 0 | 1 | 0 | 6 |
| (Rosas et al., 2015) | 1 | 1 | 1 | 0 | 1 | 1 | 1 | 1 | 1 | 8 |
| Koniak-Griffin et al., 2015) | 1 | 1 | 1 | 1 | 1 | 1 | 1 | 1 | 0 | 8 |
| (Cruz et al., 2014) | 1 | 1 | 1 | 1 | 1 | 1 | 0 | 1 | 0 | 7 |
| (Anderson et al., 2014) | 1 | 1 | 1 | 0 | 1 | 1 | 1 | 1 | 1 | 8 |
| (Nicholson et al., 2011) | 1 | 1 | 1 | 1 | 0 | 1 | 0 | 1 | 1 | 7 |
| (Vincent et al., 2013) | 1 | 1 | 1 | 0 | 1 | 1 | 1 | 1 | 0 | 7 |
| (Boudreau et al., 2013) | 1 | 1 | 1 | 1 | 1 | 1 | 1 | 0 | 1 | 8 |
| (Warner et al., 2013) | 1 | 1 | 1 | 0 | 1 | 1 | 1 | 1 | 0 | 7 |
| (Kumanyika et al., 2005) | 1 | 1 | 1 | 0 | 1 | 1 | 1 | 1 | 1 | 8 |
| (Lindsay et al., 2021) | 1 | 1 | 1 | 0 | 1 | 1 | 1 | 1 | 0 | 7 |
| (Marshall et al., 2021) | 1 | 0 | 1 | NA | NA | NA | 1 | 1 | 0 | 4 |

Yes=1, No=0, Cannot tell=0, High=7-9, Medium=4-6, Low=0-3

NA=Not applicable

Anderson, L. M., Symoniak, E. D., & Epstein, L. H. (2014). A randomized pilot trial of an integrated school–worksite weight control program. *Health Psychology*, *33*(11), 1421.

Bernstein, R., Schneider, R., Welch, W., Dressel, A., DeNomie, M., Kusch, J., & Sosa, M. (2017). Biking for health: Results of a pilot randomized controlled trial examining the impact of a bicycling intervention on lower-income adults. *WMJ: official publication of the State Medical Society of Wisconsin*, *116*(3), 154.

Boudreau, A. D. A., Kurowski, D. S., Gonzalez, W. I., Dimond, M. A., & Oreskovic, N. M. (2013). Latino families, primary care, and childhood obesity: a randomized controlled trial. *American journal of preventive medicine*, *44*(3), S247-S257.

Coday, M., Richey, P., Thomas, F., Tran, Q. T., Terrell, S. B., Tylavsky, F., Miro, D., Caufield, M., & Johnson, K. C. (2016). The recruitment experience of a randomized clinical trial to aid young adult smokers to stop smoking without weight gain with interactive technology. *Contemporary clinical trials communications*, *2*, 61-68.

Crespo, N. C., Talavera, G. A., Campbell, N. R., Shadron, L. M., Behar, A. I., Slymen, D., Ayala, G. X., Wilfley, D., & Elder, J. P. (2018). A randomized controlled trial to prevent obesity among Latino paediatric patients. *Pediatric obesity*, *13*(11), 697-704.

Cruz, T. H., Davis, S. M., FitzGerald, C. A., Canaca, G. F., & Keane, P. C. (2014). Engagement, recruitment, and retention in a trans-community, randomized controlled trial for the prevention of obesity in rural American Indian and Hispanic children. *The journal of primary prevention*, *35*(3), 135-149.

Cui, Z., Truesdale, K. P., Robinson, T. N., Pemberton, V., French, S. A., Escarfuller, J., Casey, T. L., Hotop, A. M., Matheson, D., & Pratt, C. A. (2019). Recruitment strategies for predominantly low-income, multi-racial/ethnic children and parents to 3-year community-based intervention trials: Childhood Obesity Prevention and Treatment Research (COPTR) Consortium. *Trials*, *20*(1), 1-10.

Daly, P., Pace, T., Berg, J., Menon, U., & Szalacha, L. A. (2016). A mindful eating intervention: A theory-guided randomized anti-obesity feasibility study with adolescent Latino females. *Complementary therapies in medicine*, *28*, 22-28.

DeFrank, G., Singh, S., Mateo, K. F., Harrison, L., Rosenthal, A., Gorman, A., & Leung, M. M. (2019). Key recruitment and retention strategies for a pilot web-based intervention to decrease obesity risk among minority youth. *Pilot and Feasibility Studies*, *5*(1), 1-13.

Dressel, A., Schneider, R., DeNomie, M., Kusch, J., Welch, W., Sosa, M., Yeldell, S., Maida, T., Wineberg, J., & Holt, K. (2018). Assessing health promotion interventions: limitations of traditional research methods in community-based studies. *Health promotion practice*, *19*(4), 573-580.

Garcia, D. O., Valdez, L. A., Bell, M. L., Humphrey, K., Hingle, M., McEwen, M., & Hooker, S. P. (2018). A gender-and culturally-sensitive weight loss intervention for Hispanic males: the ANIMO randomized controlled trial pilot study protocol and recruitment methods. *Contemporary clinical trials communications*, *9*, 151-163.

Griffin, T., Sun, Y., Sidhu, M., Adab, P., Burgess, A., Collins, C., Daley, A., Entwistle, A., Frew, E., & Hardy, P. (2019). Healthy Dads, Healthy Kids UK, a weight management programme for fathers: feasibility RCT. *BMJ open*, *9*(12), e033534.

Heerman, W. J., Burgess, L. E., Escarfuller, J., Teeters, L., Slesur, L., Liu, J., Qi, A., Samuels, L. R., & Singer-Gabella, M. (2018). Competency Based Approach to Community Health (COACH): The methods of a family-centered, community-based, individually adaptive obesity randomized trial for pre-school child-parent pairs. *Contemporary Clinical Trials*, *73*, 1-7.

Kumanyika, S. K., Shults, J., Fassbender, J., Whitt, M. C., Brake, V., Kallan, M. J., Iqbal, N., & Bowman, M. A. (2005). Outpatient weight management in African-Americans: the healthy eating and lifestyle program (HELP) study. *Preventive medicine*, *41*(2), 488-502.

Lindsay, A. C., Wallington, S. F., Rabello, L. M., Alves, A. D. S. M., Arruda, C. A. M., Rocha, T. C., De Andrade, G. P., Vianna, G. V., de Souza Mezzavilla, R., & de Oliveira, M. G. (2021). Faith, family, and social networks: effective strategies for recruiting Brazilian immigrants in maternal and child health research. *Journal of Racial and Ethnic Health Disparities*, *8*, 47-59.

Lynch, E., Mack, L. J., Karavolos, K., Avery, E., Liebman, R., Keim, K. S., Glover, C. M., & Fogelfeld, L. (2017). Recruitment and baseline characteristics of participants in the Lifestyle Improvement through Food and Exercise (LIFE) study. *Journal of health care for the poor and underserved*, *28*(1), 463.

Marquez, D. X., Aguiñaga, S., Castillo, A., Hughes, S. L., Der Ananian, C., & Whitt-Glover, M. C. (2020). ¡ Ojo! What to expect in recruiting and retaining older Latinos in physical activity programs. *Translational behavioral medicine*, *10*(6), 1566-1572.

Marshall, S., Taki, S., Love, P., Laird, Y., Kearney, M., Tam, N., Baur, L. A., Rissel, C., & Wen, L. M. (2021). Feasibility of a culturally adapted early childhood obesity prevention program among migrant mothers in Australia: a mixed methods evaluation. *BMC Public Health*, *21*(1), 1-18.

Metayer, N., Boulos, R., Tovar, A., Gervis, J., Abreu, J., Hval, E., Kamins, C. L., Tofuri, K., & Economos, C. D. (2018). Recruitment of new immigrants into a randomized controlled prevention trial: the live well experience. *The journal of primary prevention*, *39*(5), 453-468.

Nicholson, L. M., Schwirian, P. M., Klein, E. G., Skybo, T., Murray-Johnson, L., Eneli, I., Boettner, B., French, G. M., & Groner, J. A. (2011). Recruitment and retention strategies in longitudinal clinical studies with low-income populations. *Contemporary Clinical Trials*, *32*(3), 353-362.

Pekmezi, D., Ainsworth, C., Joseph, R., Bray, M. S., Kvale, E., Isaac, S., Desmond, R., Meneses, K., Marcus, B., & Demark-Wahnefried, W. (2016). Rationale, design, and baseline findings from HIPP: A randomized controlled trial testing a home-based, individually-tailored physical activity print intervention for African American women in the Deep South. *Contemporary Clinical Trials*, *47*, 340-348.

Rosas, L. G., Thiyagarajan, S., Goldstein, B. A., Drieling, R. L., Romero, P. P., Ma, J., Yank, V., & Stafford, R. S. (2015). The effectiveness of two community-based weight loss strategies among obese, low-income US Latinos. *Journal of the Academy of Nutrition and Dietetics*, *115*(4), 537-550. e532.

Srivastava, G., Palmer, K. D., Ireland, K. A., McCarthy, A. C., Donovan, K. E., Manders, A. J., McDougal, J., Lenders, C. M., & Apovian, C. M. (2018). Shape-up and eat right families pilot program: Feasibility of a weight management shared medical appointment model in African-Americans with obesity at an Urban academic medical center. *Frontiers in pediatrics*, *6*, 101.

Vincent, D., McEwen, M. M., Hepworth, J. T., & Stump, C. S. (2013). Challenges and success of recruiting and retention for a culturally tailored diabetes prevention program for adults of Mexican descent. *The Diabetes Educator*, *39*(2), 222-230.

Warner, E. T., Glasgow, R. E., Emmons, K. M., Bennett, G. G., Askew, S., Rosner, B., & Colditz, G. A. (2013). Recruitment and retention of participants in a pragmatic randomized intervention trial at three community health clinics: results and lessons learned. *BMC Public Health*, *13*(1), 1-12.
