# Supplementary material for: A systematic review of recruitment and retention of ethnic minorities and migrants in obesity prevention randomised controlled trials
Source: Int J Obes (Lond). 2024 Jun 4;48(8):1065–79. doi: 10.1038/s41366-024-01545-z (PMC11281904; doi:10.1038/s41366-024-01545-z)
Supplement: Supplementary file 3 — Risk of bias assessment [file 41366_2024_1545_MOESM3_ESM.docx]

**Supplementary table 3**. Risk of bias assessment of included studies

| **Author and year** | **Random sequence generation** | **Allocation concealment** | **Selective reporting** | **Blinding (participants and personnel)** | **Blinding (outcome assessment)** | **Incomplete outcome data** |
| --- | --- | --- | --- | --- | --- | --- |
| (Marquez et al., 2020) | Low | Unclear | Low | Unclear | Low | Unclear |
| (Griffin et al., 2019) | Low | Low | Low | Low | Unclear | Low |
| (DeFrank et al., 2019) | Low | Low | Low | High | Low | Low |
| (Cui et al., 2019) | Low | Low | High | Unclear | Low | Low |
| (Srivastava et al., 2018) | NA | NA | Unclear | Low | Low | Low |
| (Metayer et al., 2018) | Low | Unclear | Low | Unclear | Low | Low |
| (Heerman et al., 2018) | Low | Low | Low | Low | Low | Low |
| (Dressel et al., 2018) | Unclear | Unclear | Low | High | Low | Low |
| (Crespo et al., 2018) | Low | Low | Low | Low | Low | Low |
| (Lynch et al., 2017) | Low | Low | Low | Low | Low | Low |
| (Bernstein et al., 2017) | Low | Low | Low | High | High | Low |
| (Pekmezi et al., 2016) | Low | Low | Unclear | Low | Low | Low |
| (Garcia et al., 2018) | Low | Low | Low | Unclear | Low | Low |
| (Daly et al., 2016) | Low | Low | Low | Low | Low | Low |
| (Coday et al., 2016) | Low | Low | Low | Low | Low | Unclear |
| (Rosas et al., 2015) | Low | Low | Low | Low | Low | Low |
| (Koniak-Griffin et al., 2015) | Low | Low | Low | Low | Low | Low |
| (Cruz et al., 2014) | Low | Low | Low | Unclear | Low | Low |
| (Anderson et al., 2014) | Low | Low | Low | Unclear | Unclear | Low |
| (Nicholson et al., 2011) | Low | Low | Low | Low | Low | Low |
| (Vincent et al., 2013) | Low | Low | Low | Low | Low | Low |
| (Boudreau et al., 2013) | Low | Low | Low | Low | Low | Low |
| (Warner et al., 2013) | Low | Low | Low | Low | Unclear | Low |
| (Kumanyika et al., 2005) | Low | Low | Low | Low | Low | Low |
| (Lindsay et al., 2021) | Low | Low | Low | Unclear | Low | Low |
| (Marshall et al., 2021) | NA | NA | Unclear | Low | Low | Low |
