# Supplementary material for: A systematic review of recruitment and retention of ethnic minorities and migrants in obesity prevention randomised controlled trials
Source: Int J Obes (Lond). 2024 Jun 4;48(8):1065–79. doi: 10.1038/s41366-024-01545-z (PMC11281904; doi:10.1038/s41366-024-01545-z)
Supplement: Supplementary file 4 — Factors for effective recruitment and retention [file 41366_2024_1545_MOESM4_ESM.docx]

| ***Supplementary table 4. Facilitators of effective recruitment and retention*** | | | | | | | | | | | | | | | | | | | | | |
| --- | --- | --- | --- | --- | --- | --- | --- | --- | --- | --- | --- | --- | --- | --- | --- | --- | --- | --- | --- | --- | --- |
| **Authors, Year** | **Country** | **Sample size** | **Recruit (%)** | **Retent (%)** | **Facilitators of recruitment** | | | | | | | | **Facilitators of retention** | | | | | | | | |
|  |  |  |  |  | Comm. Champions & Networks | CHWs and health staff | Bilingual marketing | Incentives | Posters & flyers | Church outreach | Mass & social media | One to one/f2f | Flexibility & convenience | Reimburse travel | Comm. & Health workers | Rapport (keeping in touch-ongoing engagement) | Post visit phone calls | Culturally sensitive | Language & culture staff | Trained staff |  |
| Marquez et al. (2020)(30) | USA | N=438 | 54.9% | 66.67% | **X** | **X** |  |  |  |  |  |  |  | **X** | **X** | **X** |  |  |  |  |  |
| Griffin et al. (2019)(51) | UK | N=90 | 61% | 63% | **X** |  |  |  |  |  |  |  | **X** |  |  |  |  |  |  |  |  |
| DeFrank et al. (2019)(31) | USA | N=89 | 62% | 84.3% |  |  |  | **X** |  |  |  |  | **X** | **X** |  |  |  |  |  | **X** |  |
| Cui et al. (2019)(32) | USA | N= 1745 | Not reported | Not reported | **X** |  |  | **X** |  |  |  |  | **X** |  | **X** | **X** |  | **X** |  |  |  |
| Srivastava et al. (2018)(33) | USA | N= 13 | 23.4% | 40% |  |  |  |  |  |  |  |  |  |  |  | **X** | **X** |  |  |  |  |
| Metayer et al. (2018)(34) | USA | N= 406 | 38% | Not reported | **X** |  |  |  | **X** | **X** | **X** |  |  |  |  | **X** |  |  |  |  |  |
| Heerman et al. (2018)(35) | USA | N=117 | Not reported | Not reported |  |  |  | **X** | **X** |  | **X** | **X** | **X** |  |  | **X** |  | **X** |  |  |  |
| Dressel et al. (2018)(36) | USA | N=49 | 77.5% | Not reported | **X** |  | **X** |  | **X** | **X** |  |  |  |  |  |  |  |  | **X** |  |  |
| Crespo et al. (2018)(37) | USA | N= 390 | 78.16% | 67.7% |  |  |  |  |  |  |  | **X** |  |  |  |  |  |  |  |  |  |
| Lynch et al. (2017)(38) | USA | N=269 | 44.6% | 78.44% |  |  |  | **X** | **X** |  |  | **X** | **X** | **X** |  |  |  |  | **X** | **X** |  |
| Bernstein et al. (2017)(39) | USA | N=49 | 94.24% | 53.06% |  | **X** |  |  |  |  |  |  | **X** |  |  |  |  |  |  |  |  |
| Pekmezi et al. (2016)(40) | USA | N= 84 | 45.41% | Not reported |  |  |  | **X** |  | **X** | **X** | **X** |  |  |  |  |  |  |  |  |  |
| Daly et al. (2016)(41) | USA | N=47 | 78.72% | 61.0% |  |  |  |  |  |  |  |  |  |  |  |  |  |  |  |  |  |
| Coday et al. (2016)(42) | USA | N=330 | 10.67% | Not reported |  |  |  |  | **X** |  | **X** |  | - **X** |  |  |  |  |  |  |  |  |
| Rosas et al. (2015)(43) | USA | N=207 | 53.0% | Not reported |  |  |  |  |  |  |  |  |  |  |  |  |  |  | **X** | **X** |  |
| Koniak-Griffin et al. (2015)(44) | USA | N=223 | 77.43% | 87.0% |  |  |  |  |  |  |  |  |  |  |  | **X** | **X** |  |  |  |  |
| Cruz et al. (2014)(45) | USA | N=1879 | Not reported | Not reported |  | **X** |  | **X** |  |  |  | **X** | **X** |  |  | **X** | **X** | **X** | **X** | **X** |  |
| Anderson et al. (2014)(46) | USA | N=38 | 82.61% | Not reported |  |  |  |  |  |  |  |  | **X** |  |  |  |  |  |  |  |  |
| Nicholson et al. (2011)(13) | USA | N=191 | Not reported | 64% |  | **X** |  | **X** |  |  |  |  | **X** |  | **X** | **X** | **X** |  |  | **X** |  |
| Vincent et al. (2013)(47) | USA | N=58 | 63.74% | 92.6% |  |  | **X** |  | **X** |  |  |  |  |  |  |  | **X** | **X** | **X** |  |  |
| Boudreau et al. (2013)(48) | USA | N=41 | 79.0% | 67.0% |  |  |  | **X** |  |  |  |  |  |  |  |  |  |  |  |  |  |
| Warner et al. (2013)(49) | USA | N=365 | 77.0% | 86.0% | **X** | **X** | **X** |  |  |  | **X** |  | **X** | **X** | **X** |  |  |  |  |  |  |
| Kumanyika et al. (2005)(50) | USA | N=237 | 54.0% | 36.71% |  |  |  |  |  |  |  |  | **X** |  |  |  | **X** | **X** |  | **X** |  |
| Marshall et al (2021) (52) | Australia | N=163 | 95% | 78% |  |  | **X** |  |  |  |  |  |  |  |  |  | **X** | **X** | **X** |  |  |
| Lindsay et al. 2021 (11) | USA | N=233 | NA | NA | **X** |  |  |  |  |  | **X** |  |  |  |  |  |  | **X** |  |  |  |
|  |  |  |  |  | **7** | **5** | **4** | **8** | **5** | **3** | **6** | **5** | 11 | 4 | 4 | 8 | 7 | **7** | 6 | 6 |  |
| Recruitment:  Community networks includes recruiting community champions, established community networks and patient referrals.  Retention:  Rapport includes ongoing engagement activities, establishing community relationships and building trust.  Language and cultural staff include having bilingual staff and staff from similar cultural background.  Trained staff includes staff who underwent continuous training, evaluations, and debriefings, and coordinated efforts between staff for the functioning of the research. | | | | | | | | | | | | | | | | | | | | | |
